# Cognitive Functioning of Low-Grade Glioma Patients with and Without Adjuvant Treatment Before and One Year After Tumor Resection

**DOI:** 10.3390/cancers18132113

**Published:** 2026-06-29

**Authors:** Eva A. van Breugel, Iris J. M. Bras, Maud J. F. Landers, Nathalie Synhaeve, Geert-Jan Rutten, Karin Gehring

**Affiliations:** 1 Department of Neurosurgery, Elisabeth-TweeSteden Hospital Tilburg, 5022 GC Tilburg, The Netherlands; i.bras@etz.nl (I.J.M.B.); m.landers@etz.nl (M.J.F.L.); g.rutten@etz.nl (G.-J.R.); k.gehring@etz.nl (K.G.); 2 Department of Cognitive Neuropsychology, Tilburg School of Social and Behavioral Sciences, Tilburg University, 5000 LE Tilburg, The Netherlands; 3 Department of Neurology, Elisabeth-TweeSteden Hospital Tilburg, 5022 GC Tilburg, The Netherlands; n.synhaeve@etz.nl; 4 Department of Mathematics and Computer Science, Technical University Eindhoven, 5612 AZ Eindhoven, The Netherlands

**Keywords:** low-grade glioma, cognition, adjuvant treatment, radiotherapy, chemotherapy

## Abstract

**Background/Objectives**: For low-grade glioma (LGG) patients, adjuvant treatment (AT) with radiotherapy and chemotherapy may adversely impact cognition. However, existing evidence is limited by methodological heterogeneity and shortcomings. This study explored the cognitive functioning of LGG patients who underwent resection with both radiotherapy and chemotherapy (AT+) or without AT (AT−), from before resection to one year after resection. **Methods**: We included patients with World Health Organization 2021 grade 2 isocitrate dehydrogenase-mutated glioma who underwent resection between 2011 and 2024. All patients completed a neuropsychological screening battery one week before (T0) and twelve months after resection (T12), measuring reaction time, attention span, information processing speed, working memory, inhibition, cognitive flexibility, and verbal fluency. We compared cognitive performance between AT+ and AT− patients at T0 and T12, as well as trajectories of cognitive functioning, at the group and individual level. **Results**: We included 60 LGG patients (M age = 38.8 years; 63.3% male). Compared to AT− patients (n = 35), AT+ patients (n = 25) were significantly older, more frequently had tumors that crossed the midline, and reported more depressive symptoms. At T0, no significant cognitive performance differences existed between AT+ and AT− patients, despite lower observed performance in the AT+ group. At T12, AT+ patients performed significantly worse than AT− patients on mean information processing speed, due to an improvement over time in the AT− group. **Conclusions**: Patients allocated to AT may show limited cognitive recovery of information processing speed up to 12 months after surgery, without pronounced effects on other cognitive functions. These findings can guide future studies into treatment-related cognitive decline of LGG patients.

## Simple Summary

Low-grade gliomas are slow-growing brain tumors that are usually treated with surgery and, in selected patients, additionally with radiotherapy and chemotherapy. Radiotherapy and chemotherapy may negatively influence cognitive abilities, but previous studies have used various methods to research this and provided inconsistent findings. In this study, we explored changes in the cognitive functioning of patients with low-grade glioma from before surgery to one year after surgery. We compared 35 patients who underwent surgery only with 25 patients who received surgery plus additional treatment (both radiotherapy and chemotherapy). While most of the tested cognitive functions remained stable in both groups, patients who received additional treatment performed more slowly on a test of information processing speed one year after surgery compared to patients without additional treatment. These findings suggest that the information processing speed of low-grade glioma patients may be sensitive to treatment effects and provide important directions for future research.

## 1. Introduction

For patients with glioma, the impact of adjuvant treatment (AT) on cognition remains debated [1,2,3,4]. Maintenance of cognitive functioning is especially important for patients with low-grade glioma (LGG; isocitrate dehydrogenase (IDH)-mutated, World Health Organization (WHO) 2021 grade 2), because these patients are relatively young and have a median survival of 10–15 years after diagnosis [5,6]. This is a substantial period during which they may experience the long-term consequences of treatment, such as the burden of cognitive impairment in daily life [7,8]. Treatment for LGG often entails surgical resection [9] and, for some, AT consisting of radiotherapy followed by chemotherapy [10]. AT is advised for patients with LGG who are at risk of worse outcomes, such as those with higher age and larger tumors [10]. Only recently, treatment with IDH inhibitors was developed to prolong the time between surgery and AT [11]. Although AT is well-established to improve progression-free survival [12,13], its effects on the cognitive functioning of patients with LGG are not well-understood [14,15].

Various studies have sought to identify the neurological and cognitive consequences of AT. Within six months after completion of radiotherapy, changes in vascular integrity, inflammation, neurogenesis, and white matter have been identified [14,16,17], reflected in accelerated neural aging [18]. Evidence also suggests that radiotherapy for LGG can result in a decline across various cognitive domains after several months or years [14,19,20,21,22,23,24,25,26]. Reported associations include information processing speed [20,21,22,27,28], attention [21,22], executive functioning [19,20,22,28], verbal fluency [24], episodic verbal memory [21,24,28], and visual memory [19,20,29], with no consistent indication that any single cognitive domain is disproportionately affected. Few studies have examined the cognitive effects of chemotherapy in LGG patients [15]. Chemotherapy has been shown to negatively affect cognition in various other cancer types [30,31,32,33], suggesting that it may have a negative effect on LGG patients as well. In LGG patients, chemotherapy has been compared to radiotherapy, with both treatment groups showing similar courses of cognitive functioning over time [34,35,36,37].

While previous studies provide some insights into the cognitive consequences of AT, contradicting results are reported, and the quality of evidence is low for several reasons [2,15,38]. First, various studies have used the mini-mental state examination (MMSE) to assess cognitive functioning [34,36,37,39], despite its lack of validity and sensitivity for detecting cognitive impairment in the glioma population [40,41]. Second, most studies have used group means only to investigate cognitive functioning, but there is a large heterogeneity in the severity and type of cognitive impairment or decline in individual LGG patients, suggesting the need for individual analyses in research [25,26,42]. Third, various studies were cross-sectional and did not account for pre-treatment cognitive functioning. However, pre-treatment cognitive functioning has consistently been associated with cognitive outcomes after treatment [15], and without accounting for pre-treatment performance, differences between groups could be wrongfully attributed to the treatment instead of pre-existing differences. Fourth, some studies have not included a control group without AT, but this is necessary, as tumor-related factors may influence cognitive functioning over time as well [38]. Moreover, a control group aids in accounting for practice effects in repeated neuropsychological testing [43]. Last, no studies have compared patients treated with resection only to patients treated with resection followed by both radiotherapy and chemotherapy, even though these are the most commonly recommended treatment protocols [10].

Taken together, previous research on the effects of AT on the cognition of LGG patients is limited by methodological shortcomings, heterogeneous study designs, and a lack of studies including both radiotherapy and chemotherapy. Therefore, the present study explored the course of cognitive functioning in LGG patients by assessing reaction time, information processing speed, attention span, working memory, inhibition, cognitive flexibility, and verbal fluency in patients treated with resection only, compared to those with resection and both localized radiotherapy and chemotherapy, from before to one year after tumor resection. We explored both group and individual changes in cognitive functioning over time while accounting for practice effects.

## 2. Materials and Methods

### 2.1. Design

Neuropsychological screenings were performed between January 2011 and March 2024 at the neurosurgery department of the Elisabeth-TweeSteden Hospital (ETZ; Tilburg, The Netherlands), and the data were retrospectively obtained. Brain tumor patients underwent neuropsychological screening batteries a week to one day before resection (T0), three months after resection (data not used in this study), and twelve months after resection (T12) as routine clinical care. Before April 2021, T12 was conducted for research purposes and, after that, as part of clinical care. A positive advice was provided for the use of these data for research by the local ethics committees (NW2020-32, METC Brabant, The Netherlands; RP472 Ethics Review Board Tilburg University).

### 2.2. Participants

Patients were included in the current analyses if they completed the neuropsychological screening battery at T0 and T12, underwent a surgical glioma resection, and received a histopathological diagnosis of WHO grade 2 IDH-mutated glioma [44]. Patients were excluded from the study if they (1) were under 18 years of age, (2) had a history (≤2 years) of psychiatric or neurological disorders, (3) had previously undergone resection for glioma, (4) lacked a basic proficiency in Dutch, (5) received radiotherapy without adjuvant chemotherapy, or (6) completed T12 at more than 18 months after resection. Participants were categorized into two groups: those who had received localized radiotherapy followed by initiation of chemotherapy before T12 (AT+) and those who had received neither adjuvant treatment before T12 (AT−). All participants provided informed consent for the use of their data for research purposes.

### 2.3. Measures and Procedure

#### 2.3.1. Clinical Data

Histopathological diagnosis, IDH mutation and 1p/19q codeletion status, tumor location, lesion side, use of anti-epileptic medication at T0, psychotropic medication at T0, or dexamethasone at T0, radiotherapy (start and end date, type, fraction and total dose, and volume), and chemotherapy (start and end date, type, dose and number of cycles) were obtained from electronic health records and from The Netherlands Cancer Registry. For tumor volume, automated glioma segmentation was performed with an nnU-Net–based convolutional neural network [45] trained on T1, T1c, and FLAIR MRI scans from the BraTS dataset [46,47]. The resulting segmentations were visually reviewed and manually refined where necessary. Tumor volume was subsequently calculated as the total volume of the segmented voxels.

#### 2.3.2. Sociodemographic Data

Age, sex, and level of education were obtained through a standardized interview at T0. The Dutch Verhage scale for completed level of education was used, and scores were classified as low (0–4), middle (5), or high (6–7) [48].

#### 2.3.3. Psychological Symptoms

The formal Dutch translation of the Hospital Anxiety and Depression Scale (HADS) was used to assess self-reported symptoms of anxiety and depression as part of the neuropsychological screening battery. The HADS is a 14-item scale, in which the scoring of each item ranges from 0 to 3. A total score of 8 or higher on the anxiety or depression subscale denotes the clinical cut-off, which indicates considerable symptoms of anxiety or depression [49].

#### 2.3.4. Cognitive Performance

Cognitive performance was assessed using the formal Dutch translation of the Central Nervous System Vital Signs (CNS VS) computerized test battery (www.cnsvs.com) and two pencil-and-paper tests. The tests were administered on a computer under the continuous supervision of a (neuro)psychologist (in training), in the clinic or via the Microsoft Teams application. Together, the neuropsychological screening battery takes 45–60 min to complete. Data from the following four CNS VS tests were used for this study: Symbol Digit Coding, Stroop I and III, and Shifting Attention Test [50]. We excluded the CNS VS Verbal and Visual Memory tests because of their limited test-retest reliability, as demonstrated in our earlier study [51], which may hamper interpretation of the results. Additionally, we selected data from the Letter Fluency task [52] and the Digit Span (Forward and Backward) test [53] for this study. A description of these tests and scores is presented in Table 1. Raw CNS VS and Digit Span test scores were converted into z-scores adjusted for age, sex, educational level, and practice effects (for T12) using data from our own Dutch normative samples [54,55]. Published norms were used to determine T-scores for Letter Fluency scores, which were converted into z-scores [52].

### 2.4. Statistical Analyses

#### 2.4.1. Patient Characteristics

Presurgical (T0) differences between AT+ and AT− patients in sociodemographic, clinical, and psychological characteristics were explored with chi-square tests of independence and independent samples t-tests or Mann-Whitney U tests.

#### 2.4.2. Presurgical Cognitive Functioning

Differences between AT+ and AT− patients in mean test performances at T0 were analyzed with independent samples t-tests or Mann-Whitney U tests. Cohen’s d was used as an effect size for independent samples t-tests, with absolute values of 0.20 indicating a small effect, 0.50 indicating a medium effect, and 0.80 indicating a large effect [56]. The r-statistic was used as the effect size for Mann-Whitney U tests, with absolute values ≤0.30 indicating small effects, 0.31–0.50 indicating medium effects, and ≥0.51 indicating large effects.

#### 2.4.3. Course of Cognitive Functioning over Time

Changes in mean neuropsychological test scores of AT+ and AT− patients over time were analyzed with a linear mixed model (LMM) for each of the seven tests. In the models, measurements at each time point (level 1) were nested in the patients (level 2). Group (AT+ or AT−) was adopted in the LMMs as the main effect and as an interaction effect with Time. Intercepts were specified as random effects, which allowed for individual estimations of the data of each patient. Random slopes were added to the model if they significantly improved model fit according to the likelihood ratio test. The Variance Components covariance type was adopted for the random effects of each model. Model parameters were estimated with the maximum likelihood (ML) algorithm. The global fits of the models were compared using the Akaike Information Criterion (AIC) and tested with likelihood ratio tests in case of a significant effect of AT. Effect sizes of the models were estimated using R^2^ [57], with R^2^ of 0.01 indicating a small effect, 0.09 a medium effect, and 0.25 a large effect [58]. In case of a significant effect, estimated marginal means were examined with pairwise t-tests for the difference in performance between T0 and T12 in the AT+ and AT− groups separately, using Cohen’s d effect size estimation.

Changes over time in the cognitive functioning of individual patients were assessed using reliable change indices (RCI). RCIs illustrate changes in cognitive performance in individual patients compared with changes in performance of healthy controls, corrected for measurement errors and practice effects [51]. Data from our normative sample of healthy individuals was used to determine the parameters for these formulae [51,54,55]. Improvement in cognitive performance was defined as an RCI score > 1.645 and decline as an RCI score < −1.645 (corresponding with a two-tailed α of 0.10, 90% confidence interval). The number of patients with improved, stable, and declined performances for each cognitive test was compared between AT+ and AT− using chi-square tests of independence. The letter fluency test was excluded from these analyses because this test was not administered in our normative sample, and no RCI formula for letter fluency has been described in the literature.

#### 2.4.4. Cognitive Functioning at Twelve Months Post-Resection

Differences between AT+ and AT− patients in mean test scores at T12 were determined with analyses of covariance (ANCOVA), using the T0 z-scores of the same test as covariates. Scores with non-normal distributions were log-transformed. Partial Eta Squared was used as ES for ANCOVA, with values ≥ 0.02 indicating small effects, ≥0.13 indicating medium effects, and ≥0.26 indicating large effects [56].

Analyses were conducted using SPSS (version 29.0, IBM Corp., Armonk, NY, USA), except for the LMM analysis and figure creation, which were performed in RStudio (version 1.2.1). For comparison of patient characteristics, the alpha level was set to 0.05. Given the aim to explore potential cognitive side effects of AT and the relatively small sample size, a conservative correction for multiple testing may increase the risk of Type II error and obscure clinically relevant adverse effects. Therefore, a less stringent approach was adopted, using the Benjamini-Hochberg (BH) correction for multiple comparisons using a false discovery rate of 0.1 for all other comparisons [59,60].

## 3. Results

### 3.1. Patient Characteristics

**Figure 1:**
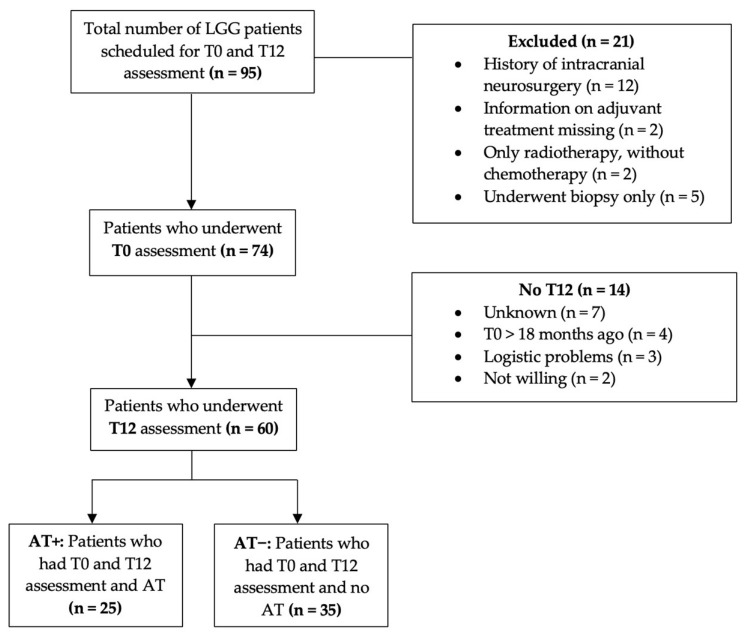
Flowchart of patient inclusion. Abbreviations: LGG = low-grade glioma; AT = adjuvant treatment.

Out of a total of 60 LGG patients who completed both T0 and T12, 25 patients underwent resection and AT with localized radiotherapy followed by chemotherapy (AT+), and 35 patients underwent resection without subsequent AT at T12 (AT−) (Figure 1).

Compared to AT− patients, AT+ patients were significantly older (*t*(58) = −3.34, *p* = 0.002), had larger tumors (*t*(29) = −2.34, *p* = 0.027), and more frequently had tumors that crossed the midline (*p* = 0.040). Additionally, AT+ patients had higher mean HADS depression scores (*t*(44) = −3.73, *p* = 0.002) and more AT+ patients scored above the cut-off (*p* = 0.010). There were no significant differences between AT+ and AT− patients on the other baseline characteristics (Table 2). Out of 25 AT+ patients, 11 (44%) received photon radiotherapy, and 14 (56%) received proton radiotherapy. Most patients underwent 28 fractions with a total dose of 50.4 Gy. By T12, all patients had completed radiotherapy, with a mean time since completion of 7.8 months. At T12, seven patients (28%) had completed or prematurely discontinued chemotherapy, while 18 patients (72%) were still undergoing chemotherapy, with a mean of 1.7 months remaining. Fifteen patients (60%) had been treated with temozolomide (TMZ), while 10 patients (40%) had received procarbazine, lomustine, and vincristine (PCV).

### 3.2. Presurgical Cognitive Functioning

At T0, the observed differences between AT+ and AT− patients on mean tests performances (Table 3) were not statistically significant (BH-corrected α = 0.014).

### 3.3. Course of Cognitive Functioning over Time

**Figure 2:**
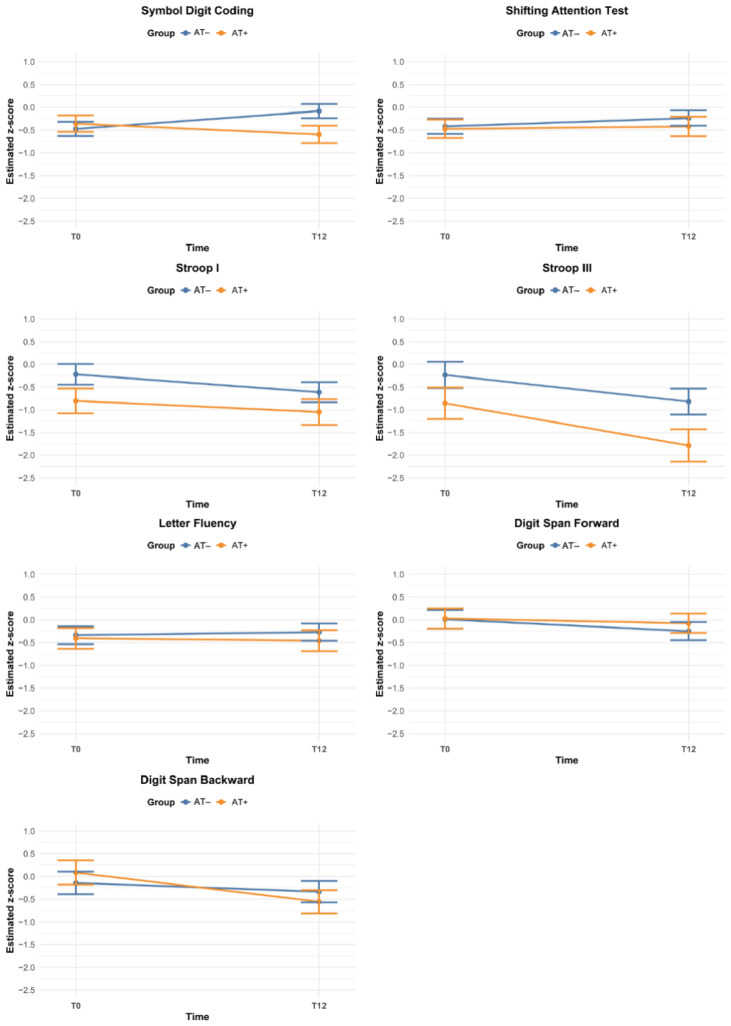
Course of observed mean group performance from T0 to T12. Bars represent standard errors for AT− (blue) versus AT+ (orange) groups. Abbreviations: AT = adjuvant treatment.

LMM analysis of the Symbol Digit Coding test score showed a significant main effect of time (*t*(52) = 2.81, *p* = 0.007), indicating an improvement from before surgery to twelve months after surgery (independent of group), as well as a significant interaction between Time and Group (*t*(52) = −2.85, *p* = 0.006), indicating a different trajectory over time between AT+ and AT− patients (Figure 2; Appendix A). Estimated marginal means of the Symbol Digit Coding test exhibited a significant improvement of the AT− group over time (*t*(52) = −2.81, *p* = 0.007, d = −0.46), with a medium effect size, and no significant change in the AT+ group after BH-correction (*t*(52) = 1.40, *p* = 0.168, d = 0.10). There were no significant predictors or interactions in the LMMs of any of the other test scores (BH-corrected α = 0.014).

**Figure 3:**
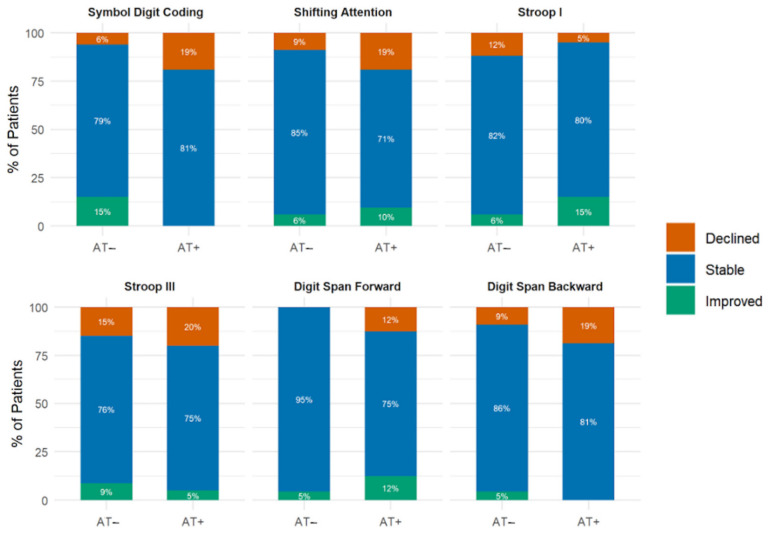
Proportions of patients with declined (orange), stable (blue), or improved (green) performance for each test between T0 and T12 according to the Reliable Change Indices (RCIs). Abbreviations: AT = adjuvant treatment.

Regarding RCI scores, there were no significant differences between AT+ and AT− patients in proportions of reliably declined, stable, or reliably improved performances over time (BH-corrected α = 0.014) (Figure 3).

### 3.4. Cognitive Functioning Twelve Months Post-Resection

At T12, AT+ patients performed significantly worse than AT− patients on the Symbol Digit Coding test (after correcting for T0 Symbol Digit Coding performance), with a medium effect size. The observed between-group differences in mean performances on the other tests were not statistically significant (BH-corrected α = 0.014) (Table 4).

**Table 4:** Comparison of estimated marginal mean z-scores at T12, corrected for T0.

	AT−		AT+				
Cognitive Test	Estimated Mean T12 z-Score ± SE	n	Estimated Mean T12 z-Score ± SE	n	F	*p* ^a^	Partial η^2^
Symbol Digit Coding	−0.03 ± 0.11	33	−0.56 ± 0.14	21	8.69	0.005 *	0.15
Shifting Attention ^b^	−0.26 ± 0.13	34	−0.38 ± 0.17	21	1.79	0.187	0.03
Stroop I	−0.70 ± 0.24	34	−1.03 ± 0.32	20	0.63	0.431	0.01
Stroop III	−1.04 ± 0.26	34	−1.50 ± 0.34	20	1.17	0.285	0.02
Letter fluency	−0.18 ± 0.16	28	−0.28 ± 0.20	18	0.16	0.695	0.00
Digit Span Forward ^b^	−0.31 ± 0.20	22	−0.03 ± 0.23	16	1.45	0.237	0.04
Digit Span Backward	−0.31 ± 0.16	22	−0.41 ± 0.19	16	0.17	0.686	0.01

^a^ Unadjusted *p*-values, BH-adjusted α = 0.011. ^b^ Scores of the Shifting Attention test and the Digit Span Forward underwent a log transformation due to non-normal distributions. Reported values were back-transformed for interpretability. * Statistically significant *p* < 0.011. Abbreviations: AT = adjuvant treatment; SE = standard error.

## 4. Discussion

In this study, we explored the course of cognitive functioning of LGG patients with and without AT, before and one year after tumor resection (on average, eight months after localized radiotherapy, and with 1.7 months of chemotherapy remaining). Although there were no presurgical differences in cognitive performance between the groups, one year after resection, patients with AT performed significantly worse on a measure of information processing speed than patients without AT, with a medium effect size. While the group without AT showed a significant improvement in information processing speed, the group with AT did not show a significant change over time, suggesting that patients who receive AT may show limited postoperative recovery.

Before resection, patients who would receive AT were older, had a larger tumor, more frequently had tumors that crossed the midline, and reported more depressive symptoms than patients who would not receive AT. This is consistent with the indication for AT in LGG, which is based on the estimated risk of tumor progression, influenced by factors such as older age, tumor volume, and bilateral tumor involvement [10,61]. Furthermore, depressive symptoms in the AT group may be partly explained by inflammation, oedema, or the use of dexamethasone. Depressive symptoms are also associated with tumor progression and may have contributed to the indication for AT [62,63]. In the analyses on cognitive functioning, we used age-corrected z-scores, but we did not correct for tumor volume or depressive symptoms. These inherent group differences may therefore confound interpretation of results, as, for example, depressive symptoms may also influence cognitive functioning [64]. However, as tumor volume is closely associated with the indication for AT, correcting for it would remove variance that is inherently tied to group membership, thereby obscuring clinically meaningful differences between the groups. Despite lower observed pre-treatment performances across cognitive tests in the group with AT, possibly due to higher tumor burden, there were no statistically significant differences in cognitive performance between patients with and without AT before surgery. This may be explained by limited statistical power.

Despite the relatively small sample size and inherent group differences, we found medium-sized differences between patients with and without AT in information processing speed over time. The distinct trajectories in information processing speed were also reflected in the frequencies of individual reliable change categories. Patients without AT showed mostly stable performance or improvement, while patients with AT showed only stable performance or decline. Reductions in information processing speed after AT have been reported in previous studies, with follow-up intervals after AT ranging from several months to multiple years [20,21,22,27,28]. Notably, the group difference in information processing speed in this study emerged at eight months after localized radiotherapy, earlier than the delayed cognitive effects previously reported [2,20,21,22,23,43,65]. Twelve months after resection, most patients were still undergoing chemotherapy, with an average of 1.7 months of treatment remaining. Therefore, the group difference in information processing speed may also reflect an acute treatment effect of chemotherapy. Moreover, the observed group difference was not driven by a decline in the AT group but by an improvement in the group without AT. This suggests that patients with AT may have more limited postoperative cognitive recovery and neuroplasticity, possibly through white matter damage in distributed information processing pathways [14,16,27,66,67,68]. These distributed pathways may be particularly sensitive to the treatment-related changes in vascular integrity, inflammation, and myelination [14,16,17]. Therefore, the lack of improvement in the group with AT could also be an early indicator of the later decline, as reported in other studies. Nevertheless, some previous studies did not detect an association between AT and information processing speed [24,29,69], potentially because these did not account for practice effects, pre-treatment performance, or lacked comparison to a group without AT.

We did not observe differences between the groups with and without AT on tests of simple reaction time, attention span, working memory, inhibition, cognitive flexibility, and verbal fluency. Previous studies, however, have reported associations between AT and impairments in executive functioning [19,20,22,28], attention [21,22], and verbal fluency [24]. These discrepancies may reflect small sample sizes, resulting in heterogeneous samples across studies. Cognitive impairment profiles vary substantially among glioma patients [25,26], and differences in treatment location [15] may contribute to variability in cognitive outcomes in this population as well. However, neither cognitive impairment profiles nor treatment location was differentiated in previous nor in the current analyses to preserve power, potentially explaining inconsistencies between studies. Notably, although group differences were not statistically significant, patients with AT observationally showed lower performance and more individual declines over time than patients without AT on most tests at both timepoints. The lack of statistically significant group differences on these tests may reflect substantial heterogeneity in cognitive functioning among patients. While such variability is clinically meaningful and may reflect differences in tumor characteristics and individual recovery patterns, it also reduces the ability to detect subtle between-group differences. In contrast, performance on information processing speed was less heterogeneous, and symbol substitution tests are highly sensitive measures of information processing speed [40], which may partly explain why a significant group difference was found for this test but not for the others.

The findings of this study contribute to the literature on the consequences of AT for LGG patients, with several strengths compared to prior studies. First, we used z-scores that were corrected for demographics and practice effects, thereby reducing potential over- or underestimations of cognitive performances over time [43]. Second, we examined both group-level averages and individual trajectories over time, providing a more complete understanding of treatment effects in a heterogeneous sample of LGG patients [42].

Certain study limitations must also be acknowledged. First, the relatively small sample size reduces statistical power, and results should be interpreted as exploratory, with limited generalizability. Moreover, given the limited sample size, we did not differentiate between types of radiotherapy and chemotherapy, treatment locations, tumor characteristics, or cognitive profiles in order to preserve statistical power. Nevertheless, collecting larger longitudinal cohorts in this population remains challenging, given the low incidence of LGG [70]. Second, the non-randomized design introduced a selection bias. The groups inherently differed at baseline because the indication for AT is based on clinical risk factors, which restricted comparability. Although we used demographically corrected z-scores and accounted for baseline cognitive functioning, residual confounding due to depressive symptoms or tumor characteristics cannot be (entirely) excluded, and results should be interpreted with caution. Moreover, as tumor burden is higher in the group receiving AT, the tumor itself may also have affected cognitive functioning differently in the groups, as it possibly did at baseline [21,38]. At the same time, the findings reflect real-world clinical practice, and it remains challenging to distinguish tumor-related effects on cognition from treatment-related effects in non-randomized research. Third, our approach prioritized breadth across multiple cognitive domains with seven tests due to the lack of consensus in prior literature and the exploratory nature of the current study. To account for these multiple comparisons, we applied a correction with a relatively lenient false discovery rate threshold. This approach balanced the risk of false positives due to multiple testing with the risk of false negatives due to the small sample size. This implies that there may be a higher risk of false positives in the current study compared to more strict multiple comparison corrections. Nevertheless, we prioritized minimizing the risk of false negatives so that clinically relevant adverse effects were not obscured while still applying a correction. Fourth, we conducted cognitive screenings at fixed time points relative to resection rather than AT. Because most patients were still undergoing chemotherapy and the interval between AT and cognitive screening varied between patients, we could not clearly distinguish the acute from the more persistent treatment effects. Studies with a fixed interval around AT or with a longer follow-up are required to distinguish transient from persistent effects. Fifth, we excluded verbal and visual memory tests from our analyses because of the limited test-retest reliability found in our previous study [51]. Nevertheless, declines in both verbal and visual memory have been associated with AT in previous studies [19,20,21,24,28,29]. Future studies may benefit from including sensitive measures of verbal and visual memory to broaden the assessment of cognitive functioning.

This exploratory study suggests that LGG patients who are allocated to AT may have a distinct cognitive trajectory from patients without AT, with the most pronounced differences observed in information processing speed. Although the beneficial effect of AT on progression-free survival is well-established, these possible distinct trajectories may be relevant in treatment planning depending on patient preferences [71]. For example, preliminary evidence suggests that by delaying AT, through IDH inhibitors, for example [11], patients may have a greater opportunity for postoperative neural and functional reorganization, leading to favorable cognitive outcomes [15]. Nevertheless, further research is needed to clarify the cognitive effects of both radiotherapy and chemotherapy in LGG patients. Multicenter collaborations could facilitate larger sample sizes in this rare population, allowing for more comprehensive testing and greater power. Moreover, studies integrating neuroimaging biomarkers with cognitive assessment may provide additional insights into the mechanisms underlying the treatment effects of AT on cognition. Additionally, the scores of neuropsychological performance testing are often only modestly correlated with self-reported cognitive functioning [72,73]. Given the relationship between cognitive complaints and quality of life [72], future studies should integrate both performance-based and self-report measures. Furthermore, a way to capture more subtle cognitive changes and fluctuations over time could be through cognitive ecological momentary assessment. This involves brief, repeated cognitive assessments administered in patients’ daily environments, capturing real-time performance [74].

## 5. Conclusions

This study explored the cognitive trajectories of LGG patients with and without AT with localized radiotherapy and chemotherapy before and one year after tumor resection. We found significantly worse information processing speed outcomes in the AT group, suggesting that patients allocated to AT may show limited cognitive recovery up to 12 months after surgery. However, our findings are exploratory due to the non-randomized design and small sample, and we cannot distinguish tumor-related effects on cognition from treatment-related effects. Nevertheless, our findings generate clinically relevant hypotheses for future, adequately powered studies. When current literature is expanded with larger, uniform studies, evidence on the effects of treatment for LGG on cognition can be used to support balanced and individualized clinical decision-making.

## Figures and Tables

**Table 1:** Description of cognitive tests used.

Test	Domain	Content	Score
Symbol Digit Coding (CNS VS)	Information processing speed	Symbols and corresponding numbers are presented at the top of the screen. Participants must match the symbols with the corresponding numbers in the lower part of the screen. Fixed test time of 2 min.	Correct responses—incorrect responses
Stroop I (CNS VS)	Simple reaction time	Participant presses the space bar when a word is presented on the screen. Fixed total of 16 items.	Average reaction time of the correct responses
Stroop III (CNS VS)	Inhibitory control	Participant presses the space bar if the color of the word does not match the meaning of the word. Fixed total of 48 items.	Average reaction time of the correct responses
Shifting Attention Test (CNS VS)	Cognitive flexibility	Participants must match geometric objects either by shape or by color to one of two colored figures at the bottom of the screen using the left and right shift keys. The rules change at random. Fixed test time of 2 min.	Correct responses—errors
Letter Fluency	Verbal ability and executive control	Participants are instructed to name as many words as possible starting with a specific letter for 1 min (three trials: D-A-T at T0 and T12, K-O-M at T3).	Total number of words named in all three trials
Digit Span Forward	Attention span	Participants are asked to repeat a series of numbers. Every two trials, a longer sequence of numbers is presented. The test is terminated when participants make two consecutive errors.	Total number of sequences correct
Digit Span Backward	Working memory	Participants are asked to repeat a series of numbers in reverse order. Every two trials, a longer sequence of numbers is presented. The test is terminated when participants make two consecutive errors.	Total number of sequences correct

Abbreviations: CNS VS = Central Nervous System Vital Signs [50].

**Table 2:** Presurgical characteristics of AT− and AT+ patients.

	AT− (n = 35)	AT+ (n = 25)	*p*
Sex: male/female, n (%)	24 (69)/11 (31)	14 (52)/11 (48)	0.193
Age at T0 (y): mean ± SD (range)	34.6 ± 8.0 (21–54)	44.7 ± 13.5 (24–68)	0.002 *
Education (y): mean ± SD (range)	16.2 ± 2.6 (10–21)	16.0 ± 2.7 (8–20)	0.825
Histological diagnosis: astrocytoma/oligodendroglioma, n (%)	24 (68)/11 (32)	17 (68)/8 (32)	0.963
1p/19q codeletion: yes, n (%)	11 (32)	8 (33)	0.938
Tumor volume (mm^3^) **: mean ± SD (range)	42,074 ± 69,601 (5668–184,760)	78,382 ± 62,186 (10,496–220,558)	0.027 *
Lesion side: left/right/bilateral, n (%)	18 (51)/17 (48)/0 (0)	13 (52)/8 (32)/4 (16)	0.040 *
Frontal involvement: yes, n (%)	27 (77)	17 (68)	0.430
Temporal involvement: yes, n (%)	12 (34)	10 (40)	0.651
Insular involvement: yes, n (%)	6 (17)	6 (24)	0.513
Parietal involvement: yes, n (%)	2 (6)	2 (8)	1.000
Occipital involvement: yes, n (%)	1 (3)	3 (12)	0.298
Awake resection: yes, n (%)	17 (49)	12 (48)	0.965
Anti-epileptic medication (T0): yes, n (%)	24 (69)	19 (76)	0.529
Dexamethasone use (T0): yes, n (%)	6 (17)	9 (36)	0.096
Psychotropic medication (T0): yes, n (%)	8 (23)	11 (44)	0.083
HADS *** anxiety (T0): mean ± SD (range)	5.9 ± 3.3 (0–14)	7.5 ± 3.7 (1–14)	0.144
Above clinical cut-off: yes, n (%)	9 (31)	9 (53)	0.142
HADS *** depression (T0): mean ± SD (range)	3.4 ± 2.7 (0–11)	6.6 ± 3.9 (1–15)	0.002 *
Above clinical cut-off: yes, n (%)	3 (10)	8 (47)	0.010 *
Radiotherapy			
Radiotherapy type: photon/proton, n (%)		11 (44)/14 (56)	
Radiotherapy number of fractions: 25/28/33, n (%)		1 (4)/22 (88)/2 (8)	
Radiotherapy total dose: 45 Gy/50.4 Gy/59.4 Gy, n (%)		1 (4)/22 (88)/2 (8)	
Time since start radiotherapy at T12 (months): mean ± SD (range)		9.0 ± 2.3 (5–14)	
Time since end radiotherapy at T12 (months): mean ± SD (range)		7.8 ± 2.3 (4–13)	
Patients who had completed radiotherapy at T12, n (%)		25 (100)	
Chemotherapy			
Chemotherapy type: TMZ/PCV, n (%)		15 (60)/10 (40)	
Time since start chemotherapy at T12 (months): mean ± SD (range)		6.6 ± 2.3 (3–12)	
Time since end chemotherapy at T12 (months): mean ± SD (range)		−1.7 ± 3.2 (−7–5)	
Patients who had completed chemotherapy at T12, n (%)		1 (4)	
Patients who prematurely discontinued chemotherapy at T12, n (%)		6 (24)	

* Significant *p* < 0.05. ** Tumor volume missing for seven patients (2 AT−, 5 AT+). *** HADS data missing for 14 patients (6 AT−, 8 AT+). Abbreviations: AT = adjuvant treatment; SD = standard deviation; HADS = hospital anxiety and depression scale; TMZ = temozolomide; PCV = Procarbazine, lomustine, and vincristine.

**Table 3:** Comparison of mean performance at T0.

	AT−		AT+				
Cognitive Test	Mean T0 z-Score ± SD	n	Mean T0 z-Score ± SD	n	t/Z	*p* ^a^	Effect Size (Cohen’s d/r-Statistic)
Symbol Digit Coding	−0.48 ± 0.95	33	−0.36 ± 1.04	25	−0.49	0.629	−0.13
Shifting Attention	−0.43 ± 1.08	34	−0.48 ± 1.21	24	−0.02 ^b^	0.987	−0.00 ^b^
Stroop I	−0.23 ± 1.01	34	−0.82 ± 1.36	23	−1.64 ^b^	0.100	−0.22 ^b^
Stroop III	−0.25 ± 1.33	34	−0.89 ± 1.62	23	−1.84 ^b^	0.066	−0.24 ^b^
Letter fluency	−0.29 ± 1.08	29	−0.33 ± 1.02	22	0.12	0.909	0.03
Digit Span Forward	−0.03 ± 0.93	22	0.08 ± 1.02	19	−0.34	0.737	−0.11
Digit Span Backward	−0.15 ± 1.38	22	0.16 ± 1.57	19	−0.71 ^b^	0.480	−0.11 ^b^

^a^ Unadjusted *p*-values, BH-adjusted α = 0.011. ^b^ Cognitive tests for which the Mann-Whitney U test was used: z-score and r-statistic. Abbreviations: AT = adjuvant treatment; SD = standard deviation.

## Data Availability

The data that support the findings of this study are not publicly available, as participants did not give written consent for data sharing and the privacy restrictions associated with the data.

## Abbreviations

The following abbreviations are used in this manuscript:

LGG	Low-grade glioma
IDH	Isocitrate dehydrogenase
WHO	World Health Organization
AT	Adjuvant treatment
MMSE	Mini-Mental State Examination
ETZ	Elisabeth-TweeSteden Hospital
HADS	Hospital Anxiety and Depression Scale
CNS VS	Central Nervous System Vital Signs
ANCOVA	Analysis of covariance
LMM	Linear mixed model
ML	Maximum likelihood
AIC	Akaike Information Criterion
RCI	Reliable change index
BH	Benjamini-Hochberg
TMZ	Temozolomide
PCV	Procarbazine, lomustine, and vincristine
RT	Radiotherapy
ChT	Chemotherapy
SD	Standard deviation
SE	Standard error

## Appendix A

**Table A1:** Results of the Linear Mixed Model (LMM) analysis of the mean course of functioning between T0 and T12 (Time) of patients with and without adjuvant treatment (Group).

Cognitive Test	AIC	Time ^a^		Group ^b^		Time × Group		R^2^
		b (SE)	*p*	b (SE)	*p*	b (SE)	*p*	
Symbol Digit Coding	284.75	0.39 (0.14)	0.007 *	0.12 (0.24)	0.631	−0.63 (0.22)	0.006 *	0.035
Shifting Attention	385.95	−0.39 (0.27)	0.158	−0.59 (0.35)	0.103	0.15 (0.44)	0.737	0.015
Stroop I	421.37	−0.59 (0.25)	0.024	−0.62 (0.45)	0.174	−0.34 (0.41)	0.407	0.037
Stroop III	323.12	0.18 (0.19)	0.356	−0.05 (0.26)	0.837	−0.13 (0.31)	0.684	0.004
Letter fluency	295.13	0.07 (0.16)	0.690	−0.07 (0.30)	0.816	−0.12 (0.26)	0.651	0.008
Digit Span forward	245.16	−0.26 (0.22)	0.243	0.02 (0.31)	0.954	0.16 (0.33)	0.638	0.021
Digit Span backward	274.94	−0.19 (0.26)	0.457	0.23 (0.36)	0.535	−0.45 (0.39)	0.254	0.002

^a^ Reference time = T12. ^b^ Reference group = AT+. * Significant *p*-values under BH-corrected α of 0.014. Abbreviations: AIC = Akaike Information Criterion; SE = standard error.
